# TfR1 mediated iron metabolism dysfunction as a potential therapeutic target for osteoarthritis

**DOI:** 10.1186/s13075-024-03304-x

**Published:** 2024-03-16

**Authors:** Wenchao Wang, Zhenkai Ma, Xuemin Feng, Jiabin Ren, Shengyao Sun, Yuandong Shao, Weimin Zhang, Xiaoxia Yang, Jiaming Zhang, Xingzhi Jing

**Affiliations:** 1 Department of Spine Surgery, Shandong Provincial Hospital affiliated to Shandong First Medical University, Jinan, 250000 Shandong China; 2 Department of Neurosurgery, Binzhou People’s Hospital, Binzhou, 256600 China; 3 Department of Endocrinology, Binzhou People’s Hospital, Binzhou, 256600 China; 4 Department of Spine Surgery, Binzhou Medical University Hospital, Binzhou, 256600 China; 5 Shandong First Medical University & Shandong Academy of Medical Sciences, No.6699 Qingdao Road, Jinan, 250117 China; 6 Department of Spine Surgery, Binzhou People’s Hospital, Binzhou, 256600 China; 7 Clinical Innovation & Research Center (CIRC), Shenzhen Hospital, Southern Medical University, Shenzhen, 518100 China

**Keywords:** Transferrin receptor-1 (TfR1), Iron metabolism, Mitophagy, mtDNA, cGAS/STING pathway

## Abstract

**Objective:**

Transferrin receptor-1 (TfR1) plays important roles in controlling cellular iron levels, but its role in OA pathology is unknown. Herein we aim to investigate the role of TfR1 in OA progression and its underlying mechanisms.

**Methods:**

TfR1 expression in cartilage during OA development were examined both in vivo and in vitro. Then IL-1β was used to induce chondrocytes degeneration in vitro and TfR1 siRNA was used for observing the effect of TfR1 in modulating iron homeostasis, mitochondrial function and degrading enzymes expression. Also the inhibitor of TfR1 was exploited to analyze the protective effect of TfR1 inhibition *in vivo.*

**Results:**

TfR1 is elevated in OA cartilage and contributes to OA inflammation condition. Excess iron not only results in oxidative stress damage and sensitizes chondrocytes to ferroptosis, but also triggers c-GAS/STING-mediated inflammation by promoting mitochondrial destruction and the release of mtDNA. Silencing TfR1 using TfR1 siRNA not only reduced iron content in chondrocytes and inhibited oxidative stress, but also facilitated the mitophagy process and suppressed mtDNA/cGAS/STING-mediated inflammation. Importantly, we also found that Ferstatin II, a novel and selective TfR1 inhibitor, could substantially suppress TfR1 activity both in vivo and in vitro and ameliorated cartilage degeneration.

**Conclusion:**

Our work demonstrates that TfR1 mediated iron influx plays important roles in chondrocytes degeneration and OA pathogenesis, suggesting that maintaining iron homeostasis through the targeting of TfR1 may represent a novel therapeutic strategy for the treatment of OA.

## Introduction

Osteoarthritis (OA) is the most common chronic joint disease, predominantly affecting middle-aged and elderly individuals [1]. The pathogenesis of OA is notably intricate, with aging and the resultant oxidative stress and mitochondrial malfunction serving as pivotal risk factors in its etiology and progression. Recent clinical pathology and mechanistic investigations have illuminated the significant role of abnormal iron accumulation within tissues in precipitating an imbalance in oxidative stress within the organism [2]. A recent clinical cohort study examining elderly male OA patients discerned a positive correlation between serum iron levels and OA severity. OA patients exhibited elevated serum iron levels, and radiographic assessments underscored a pronounced exacerbation of bone and joint affliction within the high serum iron group compared to their low serum iron counterparts [3]. Nonetheless, the role of iron in OA progress still remains elusive.

It is noteworthy that aberrant iron accumulation is not confined solely to hemophilic arthritis and rheumatoid arthritis, it has also been documented on the synovial membrane and cartilage surface in the context of senile arthritis and traumatic arthritis [4, 5]. Experimental intervention with iron chelators, such as deferoxamine (DFO), in OA chondrocytes has unveiled the capacity of DFO to inhibit the expression of arthritis-related proteins and inhibit the degradation of cartilage matrix [6]. Moreover, our previous studies have demonstrated that systemic iron overload could cause joint iron accumulation and joint degeneration [7]. These findings suggest a close correlation between iron overload and OA development. However, epidemiological evidence suggests that middle-aged and elderly people are generally in an iron overload state [8], but not all people will develop into OA. Additionally, our previous studies have revealed that numerous patients, despite having low serum iron levels, exhibit substantial iron pigment deposition within their intervertebral disc tissue [9]. We hypothesize that this phenomenon is linked to the regulation of iron metabolism at the cellular level. Interestingly, recent studies have found that multiple risk factors for OA, including mechanical stress, aging, inflammatory factors, and oxidative stress, can cause imbalances in iron metabolism and cellular iron overload [10].

Cellular iron homeostasis is primarily governed by regulating iron influx. In the blood stream, Fe^3+^ forms a complex with transferrin receptor-1 (TfR1), facilitating its cellular internalization into the cell body. Transferrin receptor TfR1 is a transmembrane glycoprotein widely expressed in various tissues and organs, with a pivotal role in mediating cellular iron influx and maintaining iron homeostasis within the cell [11]. Recent studies have revealed elevated TfR1 expression in patients with iron overload conditions, such as thalassemia, which mediates cellular iron overload and iron-related damage [12]. In central nervous system diseases such as Alzheimer’s disease, Parkinson’s disease, acute stroke, and tumors, the expression level of TfR1 is significantly higher than that of normal cell tissues [13]. Increased TfR1 expression leads to elevated cellular iron ion concentrations, making TfR1 a valuable target for the treatment of central nervous system diseases and tumors [14].

In our current study, we assessed the expression pattern of TfR1 in OA chondrocytes and explored its role in the pathogenesis of OA. We further investigated the underlying mechanism of TfR1-mediated iron overload in OA-related inflammation and cartilage degeneration. Additionally, we examined the potential of targeted TfR1 inhibition in ameliorating OA progression. Our research aims to enhance our understanding of the interplay between iron overload and OA, and the mechanisms through which iron contributes to the progression of OA pathogenesis.

## Materials and methods

### Primary chondrocytes isolation and culture

5-day-old male C57BL/6J mice were anesthetized and humanely euthanized by cervical dislocation. Cartilage from the knee joints was cut into small pieces and subjected to a 0.25% trypsin solution for 30 min and 0.25% type II collagenase for 4–6 h. After centrifugation and resuspension, primary chondrocytes were isolated and cultured in an incubator under conditions of 5% CO2 at 37 °C.

### Western blot assay

The cells were lysed in RIPA buffer containing a protease inhibitor cocktail for 15 min on ice, followed by centrifugation at 12,000 g. The total protein concentration was determined using the BCA protein assay kit (Beyotime, China). A total of 20 micrograms of protein was loaded onto a SDS-PAGE gel and separated. The separated proteins were then transferred onto PVDF membranes (Millipore, USA). Membranes were blocked with 5% BSA, then incubated with primary antibodies. TFR1(#ab84036) and HIF-1α(#ab179438) were purchased from Abcam. BNIP3(#3769S), IL-6 (#12,912), IL-1β(#12,242), iNOS (#sc-7271), COX2 (#4842), p65 (#8242), Phospho-p65 (#3033), SOX9 (#A00177-2) were purchased form CST. MMP3 (#17873-1-AP), COL2 (#28459-1-AP), MMP13 (#18,165- 1-AP), SLC7A11 (#26864-1-AP), GPX4 (#67763-1-Ig), cGAS(#A8335), STING(#19851-1-AP), DRP1(#12957-1-AP), MFF(#17090-1-AP), GAPDH (#10494-1-AP) were purchased from Proteintech. COL10 (#BA 2023, Boster) was purchased from Boster. Following an overnight incubation at 4 °C, the membranes were washed three times with Tris-buffered saline with Tween (TBST) and then incubated with the appropriate anti-rabbit or anti-mouse secondary antibodies for 1 h at room temperature. The signal intensity on the membranes was visualized using a Bio-Rad scanner (Bio-Rad, Hercules, CA).

### Immunofluorescence staining

Chondrocytes were seeded in a 12-well plate and cultured until 80% confluence. After fixation and permeabilization, the cells were blocked with 5% Bovine Serum Albumin (BSA) for 1 h. Subsequently, the cells were respectively treated with primary antibodies against COL2 (1:500), GPX4 (1:500), STING (1:500), and TfR1 (1:200) at 4 °C overnight. Afterward, they were treated with Cy3-conjugated goat anti-rabbit secondary antibody (#A0516, Beyotime, Shanghai, China, 1:500) for 1 h at 37 °C in the dark. The cells were then subjected to a washing step and stained with DAPI (Boster, AR1177) for 5 min.

To investigate the colocalization of mitochondria with BNIP3 and Drp1, cells were incubated with a diluted Mito-Tracker Red CMXRos solution (#C1049B, Beyotime, Shanghai, China, 1:500) in the dark at 37 °C for 30 min. After fixation and permeabilization, the cells were then incubated with BNIP3 (1:200) and Drp1 (1:200) antibodies at 4 °C overnight. Subsequently, the cells were treated with FITC-conjugated goat anti-rabbit secondary antibody (A0562, Beyotime, Shanghai, China, 1:500) at 37 °C for 1.5 h in the dark. Following a wash with PBS and labeling with DAPI, fluorescence microscopy (Axio Observer 3; Carl Zeiss) was used to capture images and detect differences in the fluorescence expression of the corresponding proteins.

### siRNA transfection

To induce the knockdown of STING and TfR1 in mouse chondrocytes, specific siRNA transfection procedures were conducted using the riboFECTTMCP kit (Ribo Bio, Ribobio Co. Ltd., Guangzhou, China). The chondrocytes were transiently transfected with 100 nM of each siRNA for 48 h following the manufacturer’s instructions. After transfection, the efficiency of silencing was confirmed via western blot analysis, and the most effective siSTING and siTfR1 were selected for subsequent analysis.

### Assessment of intracellular ROS and mitochondrial membrane potential (MMP)

Intracellular ROS production was assessed using a Reactive Oxygen Species Assay Kit (S0033, Beyotime, Shanghai, China) following the manufacturer’s instructions. Chondrocytes were washed three times with serum-free media. Subsequently, dichloro-dihydro-fluorescein diacetate (DCFH-DA) was diluted to 10 μM in serum-free medium and added to the cells for a 30-minute incubation in the dark. After washing the cells with serum-free media, they were examined using a fluorescence microscope (Axio Observer 3; Carl Zeiss).

Mitochondrial membrane potential (MMP) was evaluated using a mitochondrial membrane potential kit (#C2006, Beyotime, Shanghai, China). Briefly, after incubation with the JC-1 staining working solution for 20 min at 37 °C, CEP chondrocytes were rinsed with ice-cold JC-1 washing buffer three times. Multimeric JC-1 with high red fluorescence transitions to monomeric JC-1 with high green fluorescence, indicating the loss of MMP. The changes in MMP were captured using an inverted fluorescence microscope (Axio Observer A1; Carl Zeiss, Germany).

### Ferrous iron detection

After three washes with Hank’s Balanced Salt Solution (HBSS), the cells were stained with 1 μM FerroOrange (Dojindo, F374) in HBSS for 40 min at 37 °C. Subsequently, the cells underwent three additional washes with HBSS and were then subjected to imaging using a fluorescence microscope (Axio Observer 3; Carl Zeiss).

### PicoGreen and Mito-tracker red co-staining

For mtDNA staining, the CEP chondrocytes were stained with MitoTracker™ Red CMXRos probes (#M7512, ThermoFisher, USA, diluted at 1:5000) for 20 min, followed by 20 min of incubation in PicoGreen dsDNA Quantitation Reagent (#12641ES, Yeasen, Shanghai, China, diluted at 1:500) at 37 °C after being washed with PBS. Then, the microscope cover glasses were observed and imaged using a confocal fluorescence microscope (TCS SP8; Leica Microsystems, Biberach, Germany).

### Animal grouping and immunohistochemical assay

A total of 24 male C57BL/6 mice (8 weeks old) were randomly assigned to 3 groups (*n* = 8 per group): control group, OA group and OA + Ferristain II group(10 mg/kg/2d). The dosage and intervention conditions were performed according to a previous study [15]. The surgically-induced DMM model was established as detailed in prior protocols [7]. Cartilage tissues were subsequently decalcified, embedded, sectioned and stained with hematoxylin-eosin (H&E), performed to observe the morphology of cartilage and subchondral bone. The severity of OA was evaluated by OARSI assessment scoring system. All animal procedures were conducted in accordance with the guidelines of the Institutional Animal Care and Use Committee at Shandong Provincial Hospital, affiliated with Shandong First Medical University (Approval No. 2022 − 812).

For immunohistochemistry (IHC) staining, as previously described, after deparaffinization, rehydration, blocking, and antigen retrieval, the sections (4 μm, coronal plane) were then incubated with primary antibodies against TfR1, STING, COL2, MMP3, overnight at 4℃. Next, the sections were stained with the HRP-conjugated secondary antibody (#GB23303, Servicebio, dilution 1:200) for 30 min at room temperature followed colored with DAB and counterstained with hematoxylin. Immune positive staining of 5 fields randomly selected was quantitatively analyzed using Image Pro Plus software. All animal experiments were approved by the Animal Care Committee of Shandong Provincial Hospital affiliated to Shandong First Medical University.

### Micro-CT analysis

The samples were fixed in 4% paraformaldehyde (PFA) for 24 h. To assess alterations in the microarchitecture of the surgically modeled segment, micro-CT (Scanco Viva-CT80, Scanco Medical AG, Basserdorf, Switzerland) was then used to determine morphometric indices, such as intervertebral disc height and percent bone volume (BV/TV), from the volume of interest (VOI), with the resolution of 11.6 μm, 70kVp, and 114μA. And the three-dimensional (3D) images of the CEP were collected by built-in software.

### Statistical analysis

**Fig. 1:**
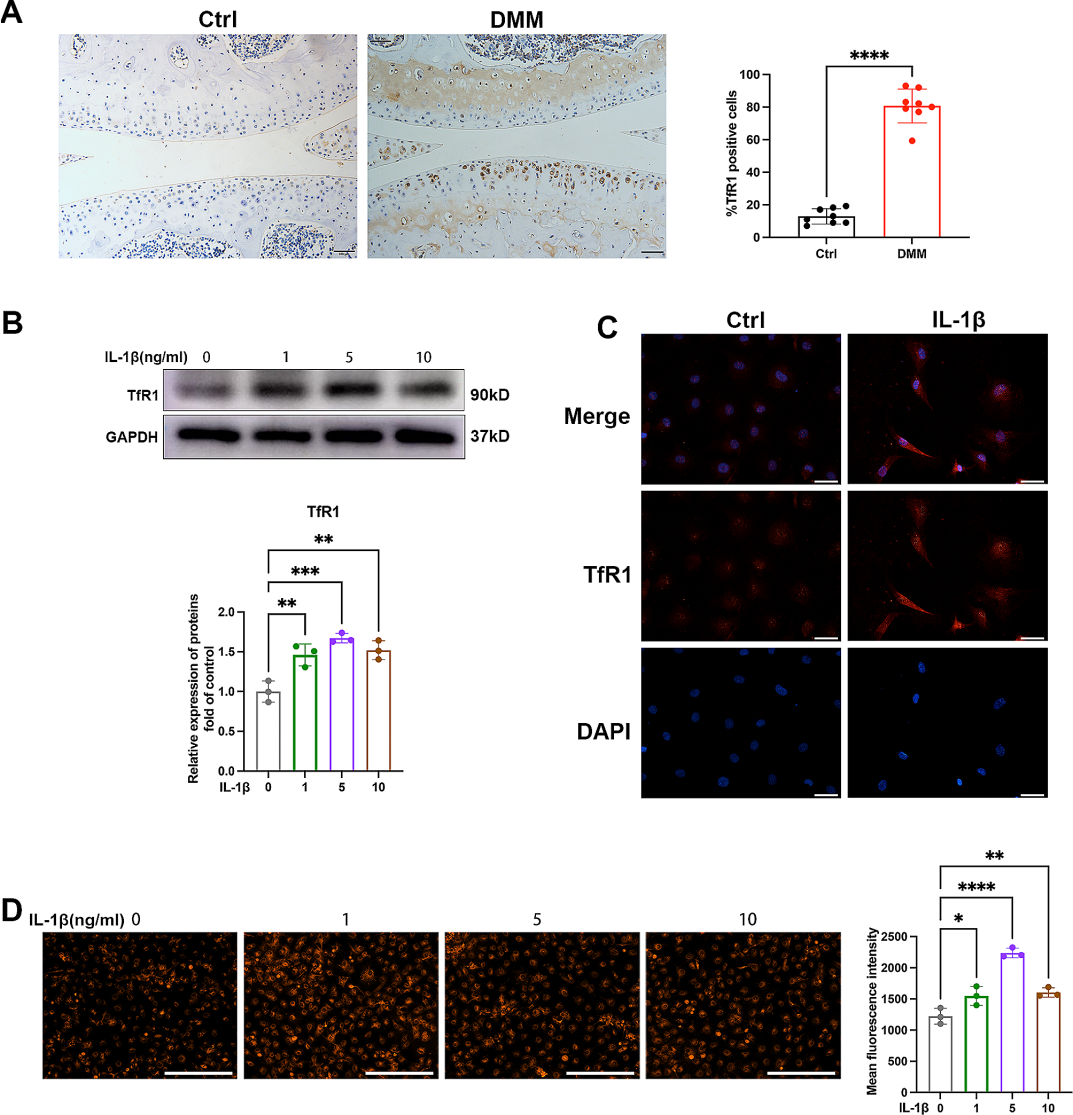
The expression of TfR1 in cartilage of OA and primary chondrocytes. (**A**) Representative images of knee articular cartilage from control group and DMM group, showing TfR1-positive chondrocytes. Scale bars = 100 μm. The ratio of TfR1 positive cells and total chondrocytes in knee articular cartilage of control group and DMM group. (*n* = 8 for each group). (**B**) The expression of TfR1 in chondrocytes treated with 0-1-5-10ng/ml IL-1β for 12 h detected by western blotting. The density of the TfR1 immune-reactive bands was analyzed by using GAPDH expression as a loading control. (**C**) Representative images of immunofluorescence staining for TfR1 expression in chondrocytes treated with IL-1β for 12 h. Scale bars = 50 μm. (**D**) Representative images for ferrous ions in the indicated group and statistical analysis of fluorescence intensity (ferrous ions). Scale bars = 200 μm. Data are presented as the mean ± SD, * *P* < 0.05, **** *P* < 0.0001

All analyses were performed with GraphPad Prism software (version 9.0; Dotmatics). Comparisons between multiple groups were analyzed using one-way ANOVA followed by Tukey’s test. For WB data and immunohistochemistry results expressed as relative fold change in Fig. 1, a Student’s t-test or a one-way ANOVA with Dunnett’s test were used for pairwise comparisons. Results are represented as mean ± SD and *p* < 0.05 was considered to be significance. All analyses were performed with GraphPad Prism software (Version 9.0).

## Results

### OA was characterized by elevated TfR1 expression and dysfunction in iron homeostasis

Firstly, we investigated the correlation between TfR1 and the progression of OA, as well as its role in regulating iron homeostasis. To do this, we conducted DMM surgery to induce a post-traumatic OA mouse model, and we observed a significant increase in TfR1-positive chondrocytes in the cartilage of the OA model compared to the sham control (Fig. 1A). Subsequently, we isolated primary murine chondrocytes and examined the expression pattern of TfR1 in vitro. As illustrated in Fig. 1B, IL-1β dose-dependently promoted TfR1 expression. Complementary immunofluorescence staining validated the increased expression of TfR1 in chondrocytes treated with IL-1β (Fig. 1C). In addition to the elevated protein expression of TfR1, iron influx increased after IL-1β treatment, as indicated by a significant increase in ferrous ions in CEP chondrocytes (Fig. 1D). These findings suggest a positive correlation between TfR1 expression and the progression of OA.

### TfR1 contributed to OA like phenotype of chondrocyte

**Fig. 2:**
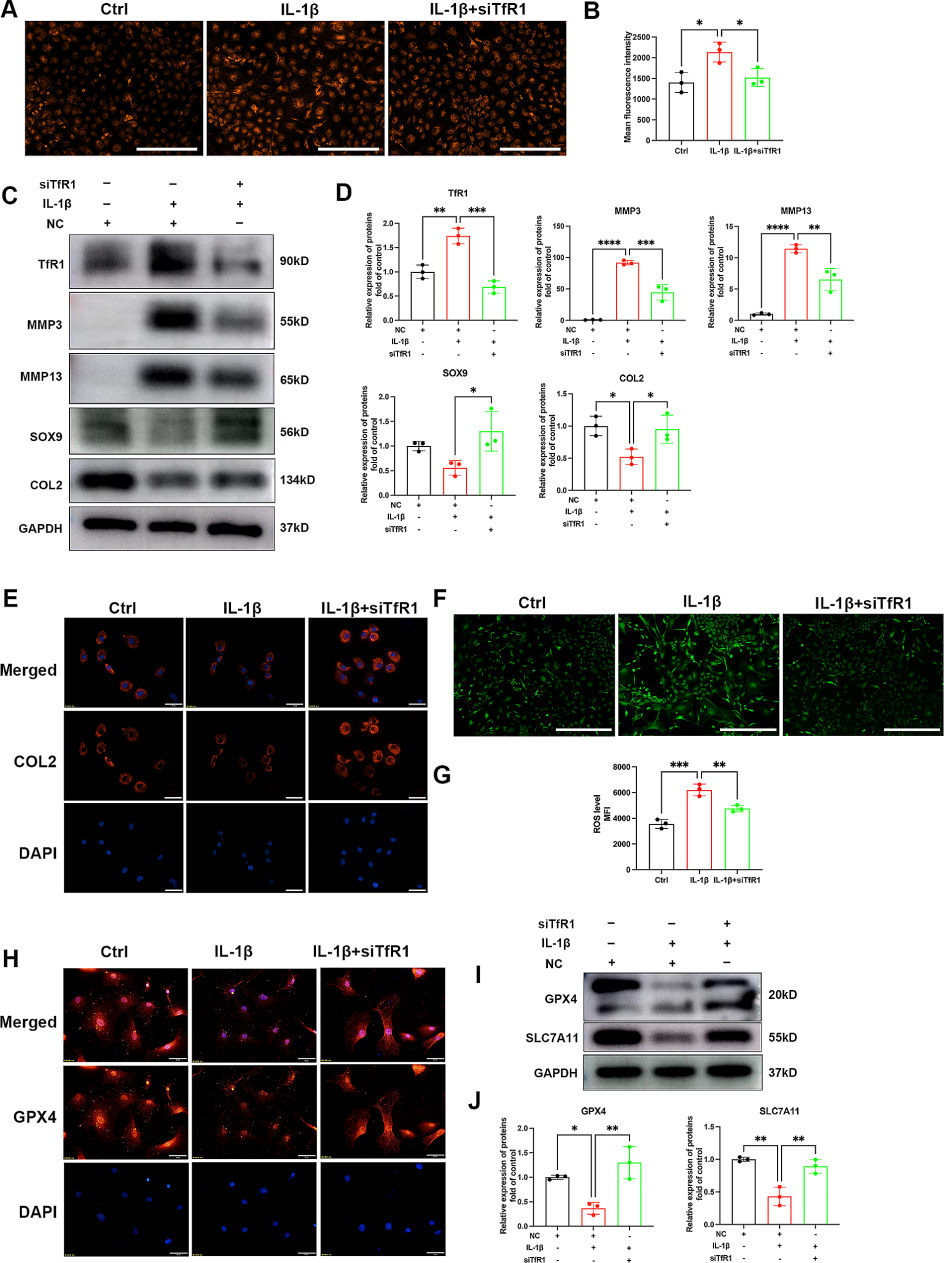
The effect of TfR1 on cartilage ECM degradation and chondrocyte ferroptosis. (**A**-**B**) Chondrocytes were treated with 5ng/ml IL-1β with or without TfR1 siRNA, representative images for ferrous ions in the indicated group and statistical analysis of fluorescence intensity. Scale bars = 200 μm. (**C**-**D**) Chondrocytes were treated with 5ng/ml IL-1β for 12 h with or without TfR1 siRNA, then expressions of TfR1, MMP3, MMP13, SOX9 and COL2 were examined by western blotting. GAPDH was included as a loading control and semi-quantitative analysis of band density was conducted. Scale bars = 200 μm. (**E**) Representative images of IF staining for COL2 expression in chondrocytes treated with IL-1β for 12 h. Scale bars = 50 μm. (**F**) Chondrocytes were treated with 5ng/ml IL-1β for 12 h, representative fluorescence microscopy photomicrographs of intracellular ROS in chondrocytes. (**G**) Flow cytometric analysis was conducted to quantify the ROS production. (**H**) Representative images of IF staining for GPX4 expression in chondrocytes treated with IL-1β for 12 h. Scale bars = 50 μm. (**I**-**J**) Expressions of GPX4 and SLC7A11 were examined by western blotting. GAPDH was included as a loading control and semi-quantitative analysis of band density was conducted. Data are presented as mean ± SD from three independent experiments. **P < 0.01, **P < 0.01, ***P < 0.001, ****P < 0.0001

We further investigated the role of TfR1 in chondrocyte ferroptosis and cartilage degeneration. TfR1 siRNA was synthesized and transfected into chondrocytes, as shown in Fig. 2A-B, TfR1 inhibition significantly reduced the concentration of iron ions in chondrocytes. Western blot analysis showed that TfR1 siRNA enhanced the SOX9 and type II collagen proteins expression, while inhibiting the expression of ECM degradation enzymes, MMP3 and MMP13 (Fig. 2C-D). Immunofluorescence staining yielded similar results, showing that TfR1 siRNA reversed the inhibition of type II collagen synthesis (Fig. 2E). Ferroptosis, a newly identified iron-dependent programmed cell death process, is highly sensitive to increased intracellular iron content. Our results revealed that TfR1 knockdown significantly inhibited excess iron induced oxidative stress, as indicated by decreased ROS production (Fig. 2F-G) and elevated expression of ferroptosis markers, including GPX4 and SLC7A11 (Fig. 2I-J). The fluorescence signal intensity of GPX4 decreased after IL-1β treatment but could be restored by TfR1 inhibition (Fig. 2H). These findings suggest that TfR1 inhibition can protect against cartilage extracellular matrix (ECM) degradation and oxidative stress induced by pro-inflammatory cytokines.

### Chondrocytes iron overload could disrupt mitochondria and activate the mtDNA/cGAS/STING pathway

**Fig. 3:**
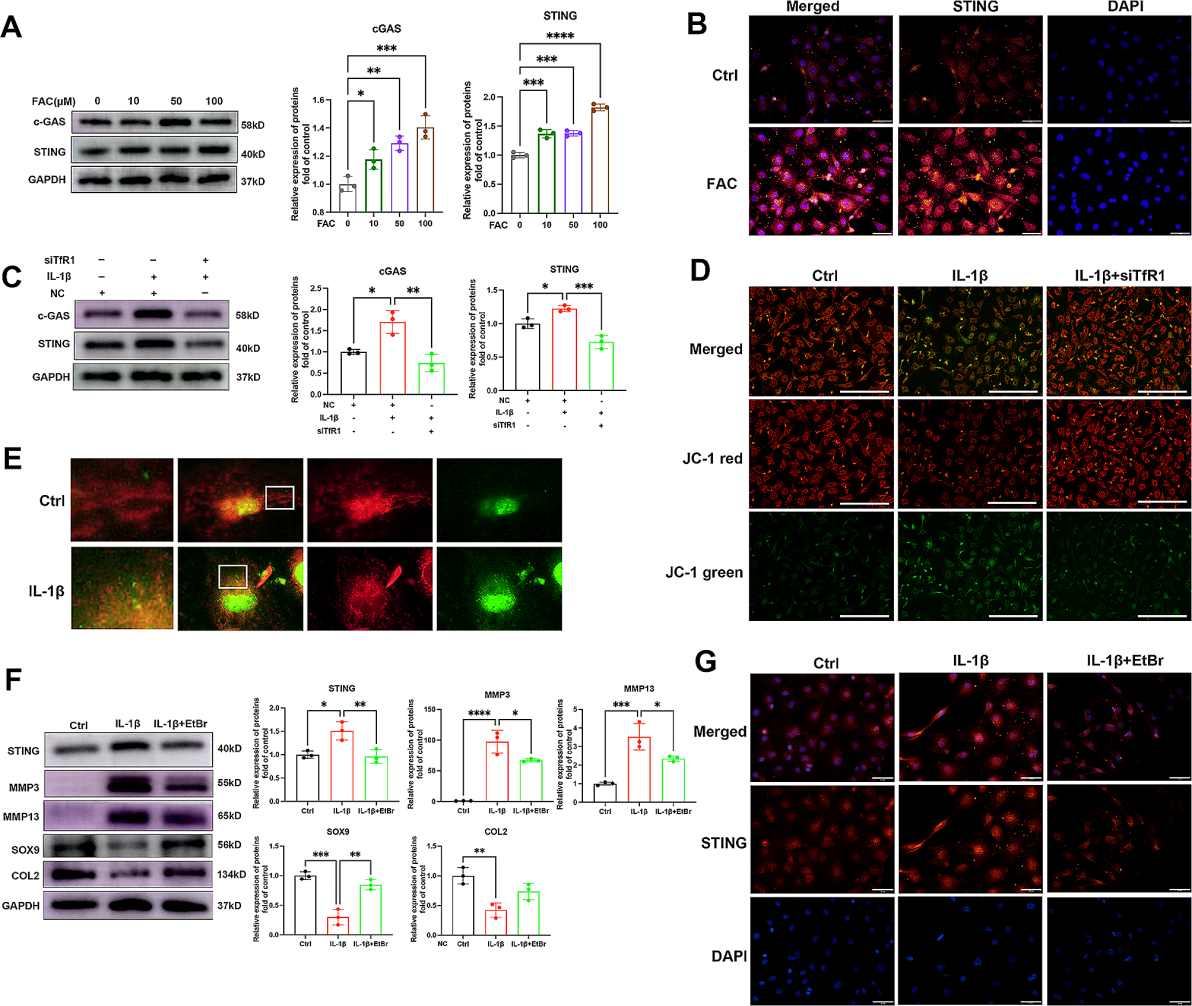
Chondrocytes iron overload could disrupt mitochondria and activate the mtDNA/cGAS/STING pathway. (**A**) Chondrocytes were treated with increasing concentrations of FAC and expressions of cGAS and STING were examined by western blotting. GAPDH was included as a loading control and semi-quantitative analysis of band density was conducted. (**B**) Representative images of IF staining for STING expression in chondrocytes treated with FAC for 24 h. Scale bars = 50 μm. (**C**) Chondrocytes were treated with 5ng/ml IL-1β for 12 h with or without TfR1 siRNA, then expressions of cGAS and STING were examined by western blotting. GAPDH was included as a loading control and semi-quantitative analysis of band density was conducted. (**D**) JC-1dye immunofluorescence staining was conducted to detect the mitochondrial membrane potential. Scale bars = 200 μm. (**E**) Representative fluorescence images of dsDNA (green) and mitochondria (red) in the control group and IL-1β treated group. (**F**) Chondrocytes were pretreated with ethidium bromide 48 h, then 5ng/ml IL-1β with or without TfR1 siRNA was added and western blot was conducted to examine STING, MMP3, MMP13, SOX9 and COL2 proteins expression. The band density was quantified and normalized to control. (**G**) Representative images of IF staining for STING expression in chondrocytes treated with IL-1β with or without EtBr. Scale bars = 50 μm. Data are presented as mean ± SD from three independent experiments. * *P* < 0.05, **P < 0.01, ***P < 0.001, ****P < 0.0001

Our prior study showed that iron overload could enhance the expression of pro-inflammatory cytokines in cartilage, including IL-6 and IL-1β [16]. However, the mechanism underlying this effect remains unclear. Recent research has demonstrated that the cGAS-STING pathway contributes to the development of various diseases by inducing inflammation, senescence, and apoptosis [17]. As illustrated in Fig. 3A, ferrous ammonium citrate (FAC) was found to promote the expression of the Stimulator of Interferon Genes (STING) protein in a dose-dependent manner. Similar results were obtained by immunofluorescence staining (Fig. 3B). IL-1β has been reported to activate cGAS-STING pathway [18]. Interestingly, our results found that TfR1 knockdown inhibited IL-1β-induced cGAS-STING activation (Fig. 3C). Mitochondria are the primary sites of cellular iron metabolism, and mitochondrial dysfunction plays a crucial role in the progression of OA [19]. Mitochondrial damage and the efflux of mitochondrial DNA (mtDNA) into the cytosol can trigger inflammatory responses through the cGAS-STING signaling pathway [20]. As depicted in Fig. 3E, treatment with IL-1β disrupted the normal morphology of mitochondria, green immunofluorescence stained dsDNA in the cytoplasm increased after IL-1β treatment, indicating that proinflammatory cytokines promoted the leakage of mtDNA from the mitochondria. TfR1 knockdown restored the normal mitochondrial morphology of chondrocytes (Fig. 3D). To further assess the role of mtDNA in stimulating STING activation in response to IL-1β, we depleted chondrocytes of mtDNA using ethidium bromide (EtBr) to inhibit mtDNA replication. Western blot and immunofluorescence staining results revealed that IL-1β-induced STING activation was inhibited (Fig. 3F-G). Moreover, EtBr inhibited IL-1β-induced ECM degradation, resulting in increased expression of SOX9 and COL2, and decreased expression of matrix enzymes MMP3 and MMP13 (Fig. 3F). These findings indicate that TfR1-mediated release of mitochondrial mtDNA and cGAS-STING pathway activation play a role in OA development.

###  STING could promote TfR1 expression and iron influx, leading to a vicious cycle

**Fig. 4:**
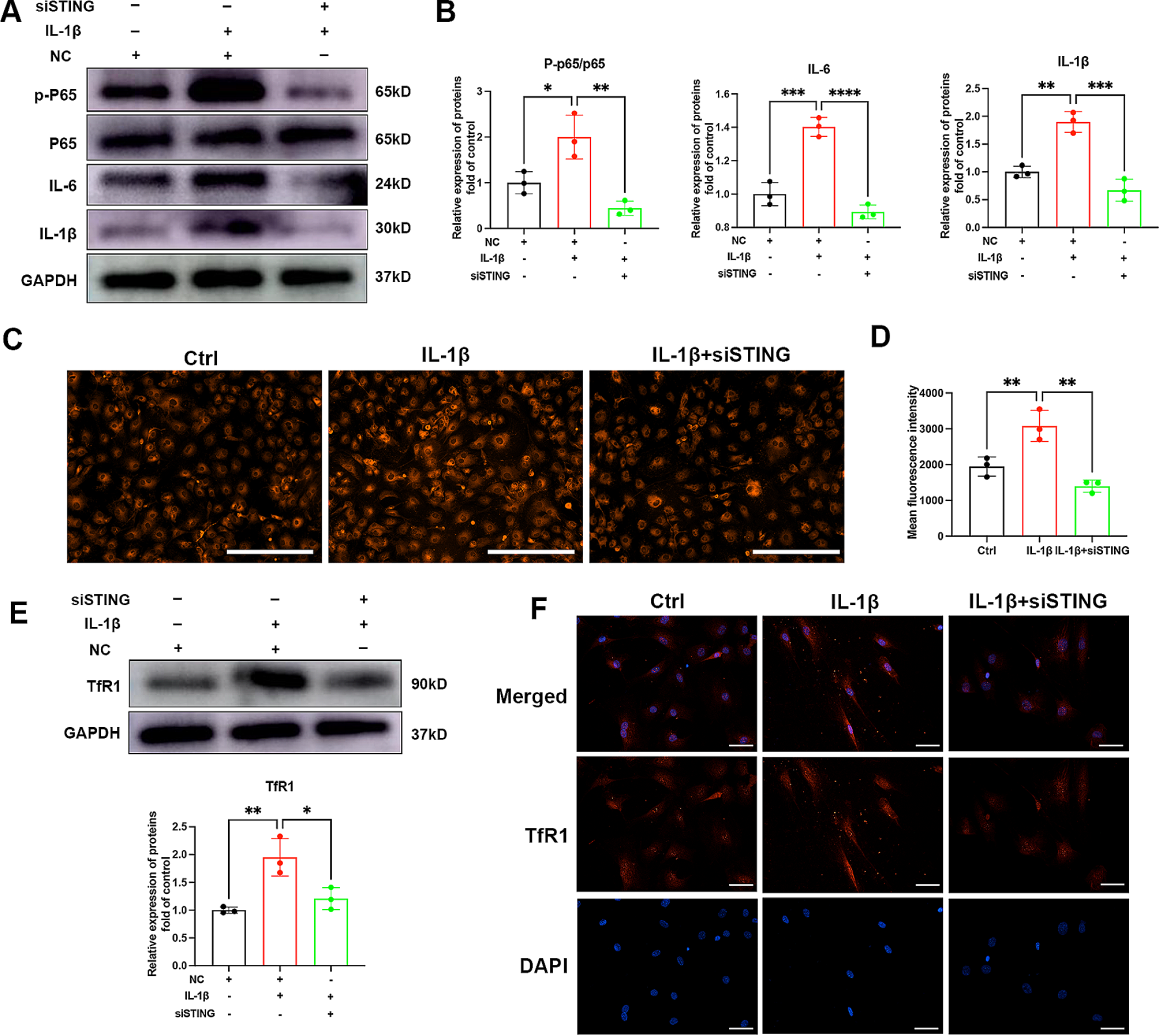
The effect of STING on chondrocytes TfR1 expression and iron influx. (**A**-**B**) Chondrocytes were treated with 5ng/ml IL-1β for 12 h with or without STING siRNA, then expressions of p-P65, P65, IL-6 and IL-1β were examined by western blotting. GAPDH was included as a loading control and semi-quantitative analysis of band density was conducted. (**C**-**D**) Chondrocytes were treated with 5ng/ml IL-1β with or without STING siRNA, representative images for ferrous ions in the indicated group and statistical analysis of fluorescence intensity. Scale bars = 200 μm. (**E**) Chondrocytes were treated with 5ng/ml IL-1β for 12 h with or without STING siRNA, then expressions of TfR1 were examined by western blotting. GAPDH was included as a loading control and semi-quantitative analysis of band density was conducted. (**F**) Representative images of IF staining for TfR1 expression in chondrocytes after STING inhibition. Scale bars = 50 μm. Data are presented as mean ± SD from three independent experiments. **P < 0.01, ***P < 0.001, ****P < 0.0001

To investigate the involvement of the cGAS-STING pathway in the expression of pro-inflammatory cytokines in OA, we synthesized STING siRNA and transfected it into chondrocytes. As illustrated in Fig. 4A-B, STING knockdown resulted in the inhibition of NF-κB signaling activation and reduced the expression of pro-inflammatory cytokines, including IL-6 and IL-1β. Interestingly, STING siRNA inhibited iron influx and reduced the concentration of iron ions (Fig. 4C-D). As indicated in Fig. 4E-F, STING knockdown also led to a decrease in chondrocyte TfR1 expression. Immunofluorescence analysis yielded similar results, showing a downregulation of the red fluorescence associated with TfR1 protein following STING inhibition. In conclusion, these results suggest that STING mediates OA inflammation and promotes iron influx through TfR1, potentially initiating a vicious cycle.

### TfR1 inhibition could activate HIF-1α/BNIP3 mediated mitophagy process

**Fig. 5:**
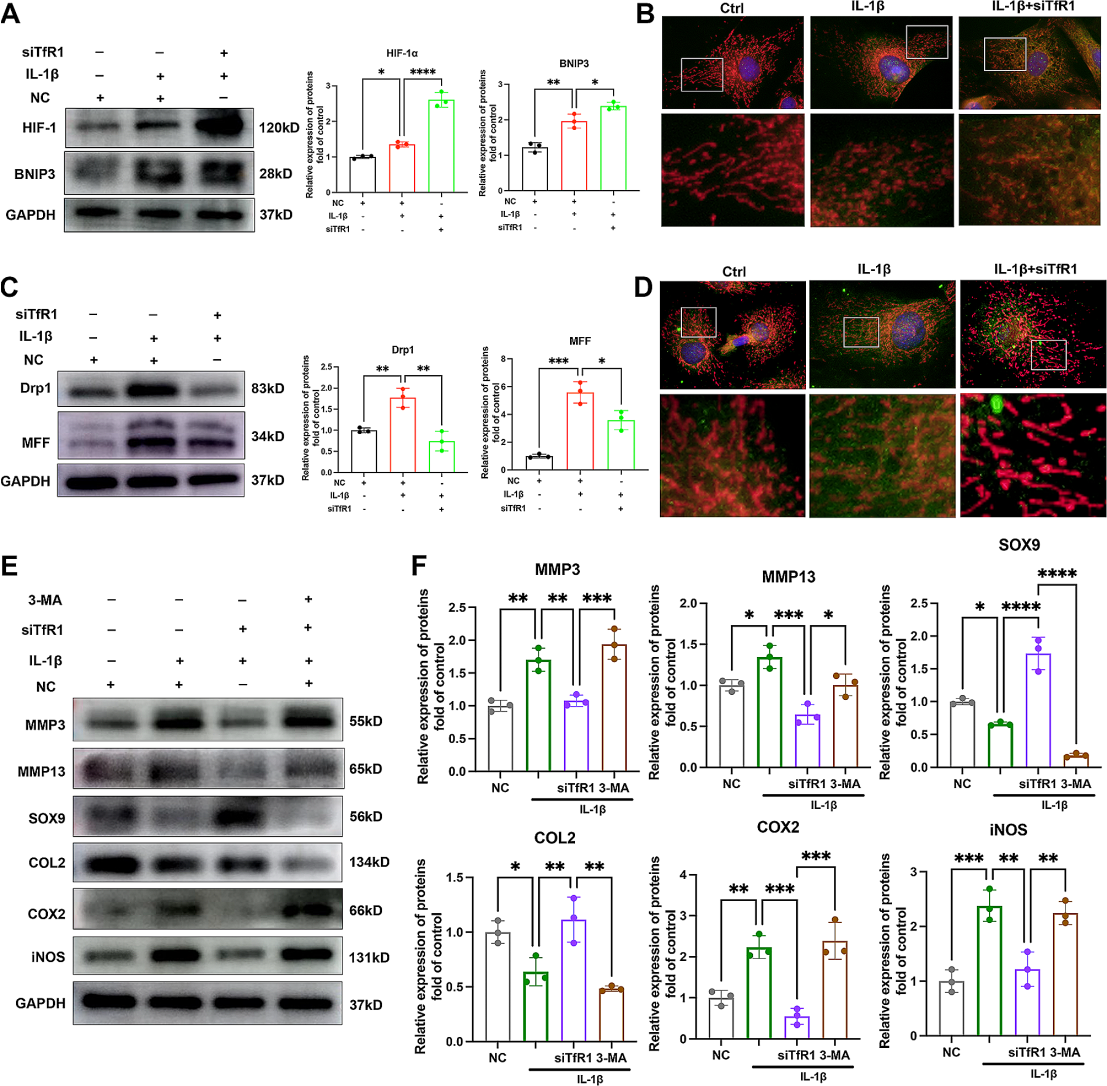
The effect of TfR1 on HIF-1α/BNIP3 mediated mitophagy process. (**A**) Representative western blotting images of HIF-1α, BNIP3 and semi-quantitative analysis of band density in IL-1β treated chondrocytes with or without TfR1 siRNA. (**B**) Representative immunofluorescence images of BNIP3. Mitochondria were stained with red mitotracker probe, BNIP3 were stained with green immunofluorescence. (**C**) Representative western blotting images of Drp-1, MFF and semi-quantitative analysis of band density in IL-1β treated chondrocytes with or without TfR1 siRNA. (**D**) Representative immunofluorescence images of Drp1. Mitochondria were stained with red mitotracker probe, BNIP3 were stained with green immunofluorescence. (**E**-**F**) Western blot analysis of SOX9, COL2, MMP13, MMP3, iNOS and COX2 expression in chondrocytes transfected with TfR1 siRNA with or without 3-MA and semi-quantitative analysis. Data are presented as mean ± SD from three independent experiments. *P < 0.05, **P < 0.01, ***P < 0.001, ****P < 0.0001

The HIF-1α/BNIP3-mediated mitophagy process plays a crucial role in eliminating dysfunctional mitochondria and is pivotal in OA development [21]. Considering the crosstalk between HIFs signaling pathway and iron metabolism, we next explored whether the protective effect of TfR1 inhibition is associated to mitophagy. As shown in Fig. 5A-B, western blot and immunofluorescence analysis showed that TfR1 knockdown promoted the expression of HIF-1α and its downstream target, BNIP3. Furthermore, TfR1 knockdown reduced the expression of mitochondrial fission proteins, including Drp-1 and MFF (Fig. 5C). Similar results were obtained with immunofluorescence staining, which showed a decrease in the co-localization of Drp-1 (green fluorescence) with mitochondria (red fluorescence) following TfR1 knockdown (Fig. 5D). To further elucidate the role of the mitophagy process in the protective effect of TfR1 inhibition in OA development, we used the autophagy inhibitor 3-MA in this study. As indicated in Figs. 5E-F, 3-MA ameliorated the protective effect of TfR1 siRNA, with down-regulated SOX9 and COL2 expression and up-regulated MMP3, MMP13, COX2, and iNOS expression. These results suggest that TfR1 inhibition could activate the HIF-1α/BNIP3-mediated mitophagy process and protect chondrocytes against degeneration through activating the mitophagy process.

### Fer-II could inhibit TfR1 expression and ameliorate OA progression

**Fig. 6:**
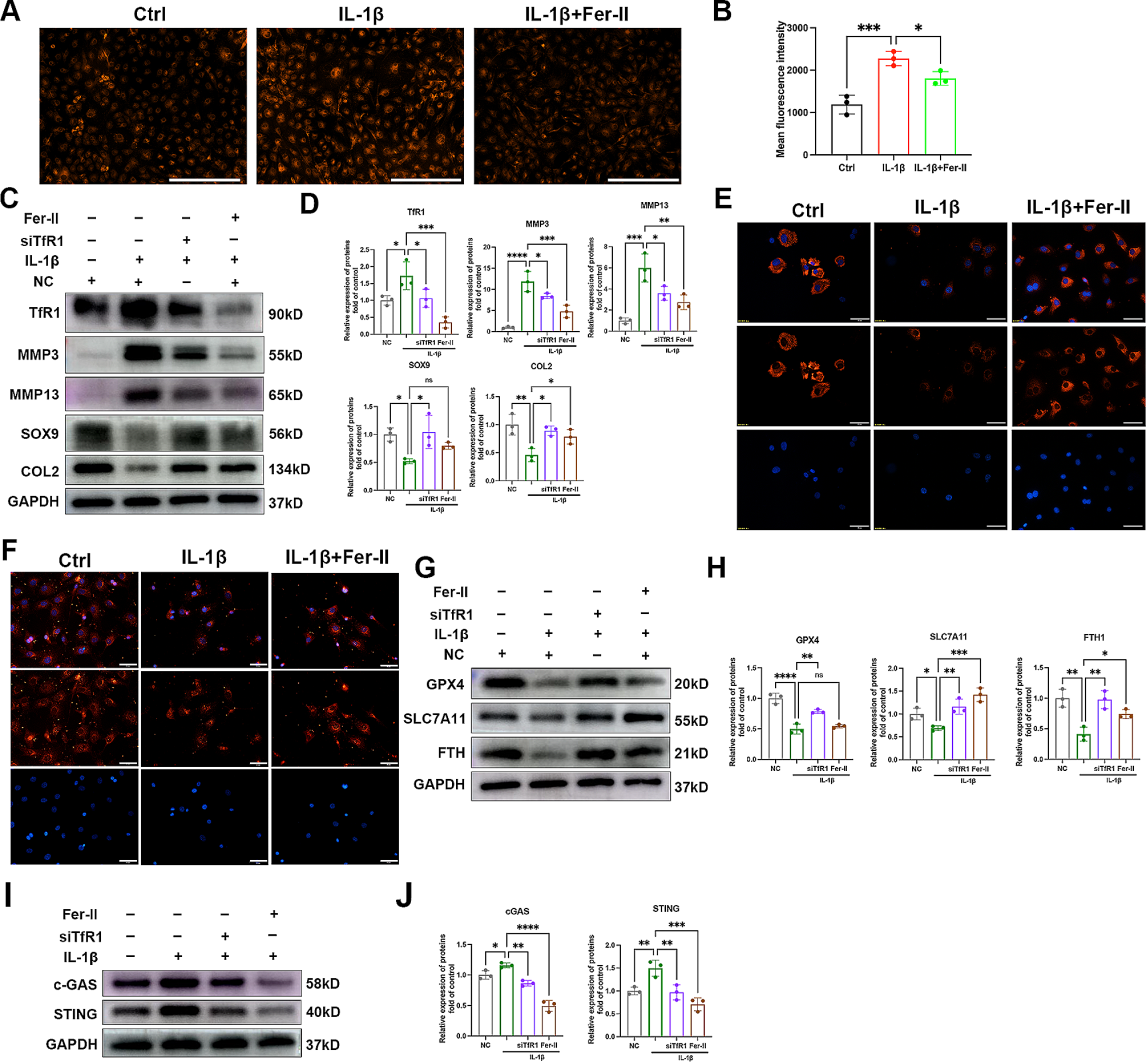
Fer-II inhibited TfR1 expression and inhibited cartilage ECM degradation. (**A**-**B**) Chondrocytes were treated with 5ng/ml IL-1β with or without TfR1 siRNA, representative images for ferrous ions in the indicated group and statistical analysis of fluorescence intensity. Scale bars = 200 μm. (**C**-**D**) Chondrocytes were treated with 5ng/ml IL-1β for 12 h with or without Fer-II, then expressions of TfR1, MMP3, MMP13, SOX9 and COL2 were examined by western blotting. GAPDH was included as a loading control and semi-quantitative analysis of band density was conducted. (**E**-**F**) Representative images of IF staining for COL2 and GPX4 expression in chondrocytes treated with IL-1β for 12 h with or without Fer-II. Scale bars = 50 μm. (**G**-**J**) Representative western blotting images of GPX4, SLC7A11, FTH, c-GAS, STING and semi-quantitative analysis of band density in IL-1β treated chondrocytes with or without Fer-II. Data are presented as mean ± SD from three independent experiments. *P < 0.05, **P < 0.01, ***P < 0.001, ****P < 0.0001

Ferstatin II, a novel and selective TfR1 inhibitor, has been shown to substantially suppress TfR1 activity in both in vivo and in vitro settings [22]. Our results demonstrated that Fer-II significantly inhibited TfR1 expression in chondrocytes and reduced the influx of iron into these cells (Fig. 6A-B). Also western blots results showed that Fer-II displayed a more pronounced protective effect in OA progression, leading to significantly higher expression levels of SOX9 and COL2, as well as lower expression of MMP3 and MMP13 (Fig. 6C-D). Immunofluorescence analysis of COL2 protein supported these findings (Fig. 6E). Furthermore, our western blot and immunofluorescence results revealed that Fer-II treatment significantly inhibited IL-1β-induced ferroptosis, as evidenced by increased levels of GPX4, SLC7A11, and FTH (Fig. 6F-H). As shown in Fig. 6I-J, we also investigated the anti-inflammatory effect of Fer-II, and our results indicated that Fer-II notably suppressed IL-1β-induced cGAS-STING activation.

**Fig. 7:**
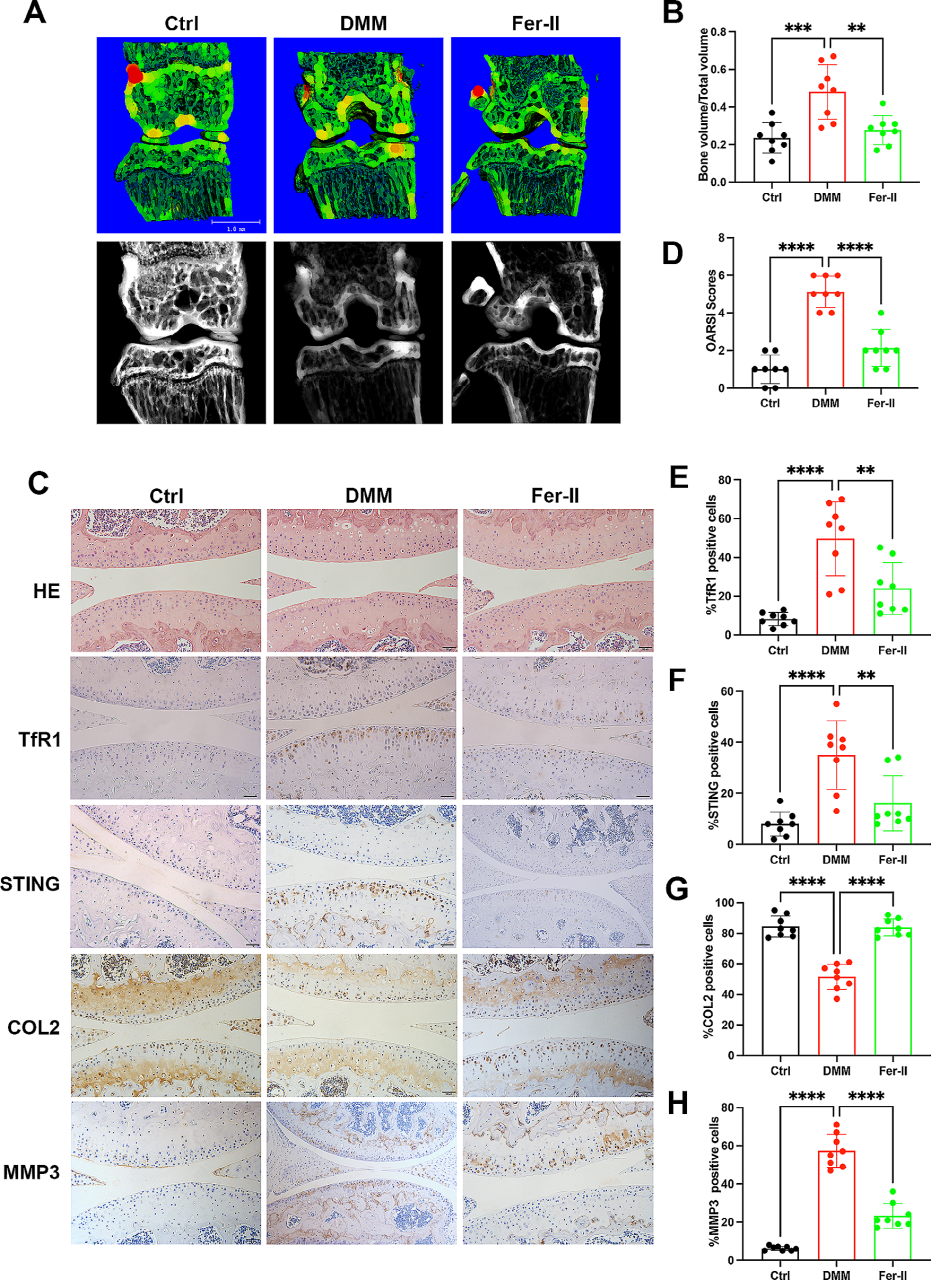
Fer-II alleviated post-traumatic OA progression. 24 mice were randomly divided in three groups and intra-articular injected Feristatin II solution followed by DMM surgery. (**A**) Micro-CT analysis, 3D reconstruction, and X-ray images of knee joints. scale bar = 1 mm. (**B**) Quantification of microarchitecture parameters [bone volume per tissue volume (BV/TV)] of the medial tibial plateau of mice knees. (**C**-**D**) HE staining of joints in the Ctrl group, DMM group, and Fer-II group and the OARSI scores. scale bar = 100 μm. (**E**-**H**) Representative IHC images and quantification of TfR1, STING, COL2, MMP3, in chondrocytes of three groups. Scale bar = 100 μm. Data are presented as mean ± SD from three independent experiments. **P < 0.01, ***P < 0.001, ****P < 0.0001

Next, we established a post-traumatic OA model through DMM surgery and proceeded to investigate the in vivo protective effect of Fer-II. Following DMM surgery, mice received intra-articular injections of Fer-II three times a week. Immunohistochemistry (IHC) results revealed that Fer-II administration significantly inhibited TfR1 expression and STING activation (Fig. 7E-F). Also Fer-II administration mitigated cartilage erosion, as indicated by a lower OARSI score in comparison to DMM mice (Fig. 7C-D). Consistently, the increased protein levels of MMP3 and the reduced expression of COL2 in the cartilage of DMM mice were substantially suppressed by Fer-II treatment (Fig. 7G-H. Additionally, we observed subchondral bone changes and osteophyte formation in these groups. The results indicated that the size of osteophytes was markedly reduced by Fer-II treatment, leading to a less obvious subchondral bone sclerosis with lower BV/TV compared to the DMM surgery group (Fig. 7A-B). In summary, these data demonstrate that the administration of Fer-II could attenuate OA development and osteophyte formation by targeting TfR1.

## Discussion

Iron overload is a common occurrence in elderly individuals and has been linked to a multitude of diseases, including Parkinson’s disease, osteoporosis, chronic renal failure, Alzheimer’s disease, and arteriosclerosis [23]. However, the mechanisms regulating iron metabolism under OA pathogenic conditions and the processes leading to iron deposition in tissues remain elusive. In the present study, we demonstrated that TfR1 is elevated in OA cartilage and plays a role in mediating iron deposition in chondrocytes. Excess iron not only results in oxidative stress damage and sensitizes chondrocytes to ferroptosis, but also triggers c-GAS/STING-mediated inflammation by promoting mitochondrial destruction and the release of mtDNA. Silencing TfR1 using TfR1 siRNA and Fer-II not only reduced iron content in chondrocytes and inhibited oxidative stress, but also suppressed mtDNA/cGAS/STING-mediated inflammation. In conclusion, our work demonstrates that maintaining iron homeostasis through the targeting of TfR1 may represent a novel therapeutic strategy for the treatment of OA.

Iron is the most abundant trace element in the human body and plays a crucial role in normal metabolic processes. Due to the absence of an efficient mechanism for iron excretion, factors such as aging, elevated dietary iron intake, multiple blood transfusions, genetic mutations, and others can lead to the accumulation of iron in the body [24]. This iron deposition in tissues has been linked to various diseases and organ degeneration, affecting areas like bones, the brain, and the kidneys [25, 26]. However, the mechanisms through which excess iron damages chondrocytes under OA pathogenic conditions remain poorly understood. In the current study, we observed a significant upregulation of TfR1 in OA chondrocytes. Pro-inflammatory cytokines were found to disrupt iron homeostasis and promote iron influx by increasing TfR1 expression. TfR1 is the primary gatekeeper for cellular iron and plays a central role in controlling iron homeostasis [27]. A previous study of ours has demonstrated that an iron-overloaded mouse model did not spontaneously develop osteoarthritis (OA) within a two-month period. However, iron overload markedly accelerated the development of OA in mice that underwent destabilization of the medial meniscus (DMM) surgery. This suggests that TfR1-mediated iron homeostasis dysfunction and continuous iron influx are prerequisites for iron overload-induced chondrocyte ferroptosis and cartilage degeneration.

OA is characterized by chronic, systemic, low-level inflammation and cartilage degeneration. Inflammation plays a pivotal role in the progression of OA [28]. An increasing body of evidence suggests that ferroptosis is often accompanied by inflammatory manifestations [29]. Ferroptotic cells release damage-associated molecular pattern molecules (DAMPs) and alarmins, which are recognized by immune cells and tissues. Pattern recognition receptors (PRPs) detect these molecules on the cell surface, thereby inducing the migration and infiltration of inflammatory cells and the release of tissue inflammatory factors [30]. Mitochondria are the primary cellular organelles for iron metabolism [31]. In our study, our results indicate that excess iron in chondrocytes can result in mitochondrial destruction and the release of mtDNA into the cytoplasm, activating the cGAS/STING pathway and promoting the expression of inflammatory mediators. Inhibiting mtDNA using EtBr significantly suppresses the cGAS/STING pathway and cartilage ECM degradation, underscoring the pivotal role of mitochondrial dysfunction in iron metabolism-induced OA progression. Furthermore, we also observed that silencing STING not only inhibited the expression of pro-inflammatory cytokines but also reduced TfR1 expression. This suggests that the activation of the cGAS/STING pathway can further enhance TfR1 expression by promoting the expression of inflammatory mediators, leading to a vicious cycle. Clinical evidence has shown that transient bleeding in the knee joint can lead to arthritis. However, after approximately a week, inflammatory factors in the knee joint return to normal levels, while hemosiderin accumulates in the cartilage and lining of the knee joint [32]. The deposition of iron in synovial tissue may be the primary cause of sustained degeneration in the knee joint.

Mitophagy is a critical cellular function that serves to eliminate damaged mitochondria, allowing cells to recycle and maintain their health. Late-stage OA cartilage exhibits a reduced level of mitophagy, resulting in excessive ROS production and ongoing mitochondrial degradation [33]. The HIF-1α/BNIP3-mediated mitophagy process has been shown to play protective roles in the progression of OA. Research has also indicated that HIF-1α is involved in the regulation of iron metabolism within hypoxic microenvironments [34]. The activity of HIF-1α is negatively regulated by prolyl hydroxylase (PHD), which is dependent on oxygen and ferrous ions. Under conditions of hypoxia or cellular iron deficiency, the activity of PHD is inhibited, leading to increased stability of HIF-1α [35]. In our experiments, TfR1 knockdown activated the HIF-1α/BNIP3 pathway and facilitated autophagosome formation. Furthermore, our findings revealed that the expression of inflammatory and catabolic mediators increased following treatment with the autophagy inhibitor 3-MA, which attenuated the protective effect of TfR1 inhibition.

Ferstatin II, a novel and selective TfR1 inhibitor, has been proven to substantially promote TfR1 degradation in vivo and in vitro. Additionally, Fer-II has demonstrated its ability to suppress ferroptosis, making it a potential therapeutic approach for managing neurological disorders [15]. In our current study, our in vitro results show that Fer-II has a more pronounced effect on inhibiting TfR1 protein expression and mitigating cartilage degeneration. Cartilage degeneration is a hallmark feature of OA, and the two primary strategies are delaying degeneration and promoting regeneration. The results from our animal experiments in this study indicate that Fer-II can ameliorate OA cartilage degeneration and promote the type II collagen expression, as evidenced by a lower OARSI score and reduced ECM loss. These in vitro and in vivo findings further validate the protective role of the TfR1 inhibitor Fer-II in OA progression.

## Conclusions

This study reveals an interaction between iron metabolism and inflammatory factors in OA pathology. Pro-inflammatory cytokines could disrupt chondrocytes iron homeostasis via promoting TfR1 expression. Excess iron in chondrocytes would disrupt mitochondrial function, resulting in mtDNA leakage and cGAS-STING activation. mtDNA-cGAS-STING mediated inflammatory factors could in turn further promote TfR1 expression, leading to a vicious cycle. Our study indicate that maintaining iron homeostasis via targeting TfR1 may be a novel therapeutic strategy for OA.

## Data Availability

No datasets were generated or analysed during the current study.

## Abbreviations

BV/TV: Bone volume/Total volume
CEP: Cartilage endplate
CT: Computed tomography
DCFH-DA: Dichloro-dihydro-fluorescein diacetate
DMM: Destabilization of the medial meniscus
ECM: Extracellular matrix
FAC: Ferrous ammonium citrate
Fer-II: Ferristatin-II
H&E: Hematoxylin-eosin
MMP: Mitochondrial membrane potential
mtDNA: Mitochondrial DNA
OA: Osteoarthritis
OARSI: Osteoarthritis Research Society International
STING: Stimulator of Interferon Genes
TfR1: Transferrin receptor-1
